# Evaluation of a Selective Chemical Probe Validates That CK2 Mediates Neuroinflammation in a Human Induced Pluripotent Stem Cell-Derived Microglial Model

**DOI:** 10.3389/fnmol.2022.824956

**Published:** 2022-06-14

**Authors:** Swati Mishra, Chizuru Kinoshita, Alison D. Axtman, Jessica E. Young

**Affiliations:** ^1^Department of Laboratory Medicine and Pathology, Seattle, WA, United States; ^2^Institute for Stem Cell and Regenerative Medicine, University of Washington, Seattle, WA, United States; ^3^Division of Chemical Biology and Medicinal Chemistry, UNC Eshelman School of Pharmacy, University of North Carolina at Chapel Hill, Chapel Hill, NC, United States

**Keywords:** Alzheimer’s disease, hiPSC models, casein kinase 2, neuroinflammation, chemical probe, SGC-CK2-1

## Abstract

Novel treatments for neurodegenerative disorders are in high demand. It is imperative that new protein targets be identified to address this need. Characterization and validation of nascent targets can be accomplished very effectively using highly specific and potent chemical probes. Human induced pluripotent stem cells (hiPSCs) provide a relevant platform for testing new compounds in disease relevant cell types. However, many recent studies utilizing this platform have focused on neuronal cells. In this study, we used hiPSC-derived microglia-like cells (MGLs) to perform side-by-side testing of a selective chemical probe, SGC-CK2-1, compared with an advanced clinical candidate, CX-4945, both targeting casein kinase 2 (CK2), one of the first kinases shown to be dysregulated in Alzheimer’s disease (AD). CK2 can mediate neuroinflammation in AD, however, its role in microglia, the innate immune cells of the central nervous system (CNS), has not been defined. We analyzed available RNA-seq data to determine the microglial expression of kinases inhibited by SGC-CK2-1 and CX-4945 with a reported role in mediating inflammation in glial cells. As proof-of-concept for using hiPSC-MGLs as a potential screening platform, we used both wild-type (WT) MGLs and MGLs harboring a mutation in presenilin-1 (PSEN1), which is causative for early-onset, familial AD (FAD). We stimulated these MGLs with pro-inflammatory lipopolysaccharides (LPS) derived from *E. coli* and observed strong inhibition of the expression and secretion of proinflammatory cytokines by simultaneous treatment with SGC-CK2-1. A direct comparison shows that SGC-CK2-1 was more effective at suppression of proinflammatory cytokines than CX-4945. Together, these results validate a selective chemical probe, SGC-CK2-1, in human microglia as a tool to reduce neuroinflammation.

## Introduction

Many neurodegenerative disorders that affect aging populations, such as Alzheimer’s disease (AD), have few effective treatments (Krahn et al., 2020). AD is characterized by accumulations of amyloid beta (Aβ) peptides in extracellular plaques, intracellular accumulations of hyperphosphorylated tau protein in neurofibrillary tangles (NFTs), synaptic loss, and neuroinflammation. Human induced pluripotent stem cell models have been increasingly utilized to study AD because brain-relevant cell types can be studied *in vitro* and used to profile molecules that may modify these disease phenotypes. Indeed, hiPSC-derived cells have been employed in several screens of small molecules hypothesized to impact AD processes, however, the majority of these experiments have been performed in neuronal cells and largely focused on the effects of compounds on Aβ and tau (Brownjohn et al., 2017; Kondo et al., 2017; van der Kant et al., 2019; Cheng et al., 2021).

One issue to consider when testing small molecules is that most commercially available compounds are suboptimal and have many off-target effects. Therefore, efforts are underway to develop high-quality small molecules to enable interrogation of biological pathways. These molecules, known as chemical probes, need to meet certain criteria relating to on-target potency, selectivity, and cell-based activity (Arrowsmith et al., 2015). Wells et al. (2021) recently developed a chemical probe for the human protein kinase CK2, which has been implicated in the phosphorylation of hundreds of cellular proteins. CK2 is a ubiquitously expressed and evolutionarily conserved pleiotropic serine/threonine kinase that regulates many essential and divergent signaling pathways (Meggio and Pinna, 2003). Previously, the CK2 chemical probe, SGC-CK2-1, outperformed all published inhibitors in terms of kinome-wide selectivity, demonstrated potent inhibition of CK2, and proved active in cells, making it a strong candidate for further studies investigating disease-specific pathways (Wells et al., 2021).

Recent evidence supports that CK2 plays essential functions in the mammalian brain (Perez et al., 2011; Castello et al., 2017; Borgo et al., 2021). Research in this area has been partially motivated by the finding that expression of CK2 is much greater in the mammalian brain when compared to other tissues (Blanquet, 2000; Castello et al., 2017). As such, CK2 has been characterized as an emerging target for neurological, psychiatric, and neurodegenerative diseases (Perez et al., 2011; Castello et al., 2017; Borgo et al., 2021). CK2 was one of the first kinases shown to be aberrant in AD (Iimoto et al., 1990). Additional recent studies have supported this finding and shown that CK2 regulates pathways involved in protein aggregation and neuroinflammation. For example, tau protein is a substrate of CK2 and hyperphosphorylation of tau by CK2 has been suggested to contribute to its resultant accumulation in NFTs (Avila et al., 1994; Rosenberger et al., 2016; Zhang et al., 2018).

In addition to the canonical pathological hallmarks of senile plaques and NFTs, neuroinflammation is apparent in AD pathology and is mediated largely through microglia, the innate immune cells of the central nervous system (Wyss-Coray and Rogers, 2012). When microglia are activated in AD they can release pro-inflammatory cytokines, such as IL-6 and IL-1β, which can exacerbate other pathologies (Hansen et al., 2018). In late stages of AD, chronic inflammation induced by microglia contributes to progressive neuronal cell death (Ransohoff and El Khoury, 2015). Dampening this chronic inflammatory response holds great potential as a disease modifying therapy for AD. CK2 may play a role in AD-specific neuroinflammation based on the findings that CK2 immunoreactive astrocytes are increased in AD vs. non-demented controls and they surround Aβ deposits (Rosenberger et al., 2016). However, little is known about how inhibiting CK2 affects microglia-mediated pathways of neuroinflammation.

Due to the role of microglia in mediating AD-related neuroinflammation and potential function of CK2 inhibition in curbing chronic neuroinflammation, we mined the current literature and publicly available RNA-seq databases to analyze neuroinflammatory-related kinases in microglia that can be inhibited by two CK2 inhibitors: SGC-CK2-1 and commercially available inhibitor, CX4945. Based on the divergent inhibitory profiles of these two compounds, we hypothesized that the selective chemical probe SGC-CK2-1 may represent a tool that can be used to specifically explore the modulation of CK2-dependent expression of proinflammatory cytokines induced in human microglia by treatment with LPS. Literature reports also suggested that inhibition of CK2 *via* SGC-CK2-1 would reduce expression of cytokine release in LPS-stimulated human microglia. hiPSC-derived MGLs respond strongly to LPS treatment and LPS was found to induce CK2 activity in murine macrophage cells (Lodie et al., 1997; Abud et al., 2017). To test our hypothesis, we differentiated MGLs from an hiPSC line engineered to harbor two copies of a penetrant FAD mutation in PSEN1, PS1ΔE9, and its isogenic WT control cell line (Woodruff et al., 2013). These cell lines have previously been shown to have gene dose-dependent effects of the ΔE9 mutation in hiPSC-derived neurons (Woodruff et al., 2013). We investigated the effect of SGC-CK2-1 vs. CX-4945 on pro-inflammatory cytokine expression in LPS-stimulated human microglia derived from hiPSCs. We examined mRNA and protein for IL-6 and IL1-β, two pro-inflammatory cytokines that are increased in AD patients and, when increased, correlate with cognitive impairments associated with AD (Forlenza et al., 2009; Huang and Sheng, 2010; Swardfager et al., 2010; Brosseron et al., 2014; Scarabino et al., 2020; Lyra et al., 2021).

## Materials and Methods

### Cell Lines

An hiPSC cell line homozygous for the point mutation in the PSEN1 gene known to cause FAD, “ΔE9/ΔE9”, and its isogenic control hiPSC cell line “WT/WT,” were generated as described previously (Woodruff et al., 2013).

### Differentiation of Human Induced Pluripotent Stem Cells Into Microglia Like Cells

WT/WT and ΔE9/ΔE9 hiPSCs were differentiated into MGLs using a previously described protocol (McQuade et al., 2018). Briefly, hiPSCs were differentiated into hematopoietic progenitor cells (HPCs) using the STEMdiff™ Hematopoietic Kit (#05310, Stem cell Technologies). HPCs were either frozen using Bambanker HRM freezing media (#BBH01; Bulldog Bio) or further differentiated into MGLs. HPCs were cultured in microglia differentiation medium comprised of DMEM/F12 (#11039047; Thermo Fisher Scientific), B27 (#17504-044; Thermo Fisher Scientific), N2 (#17502-048; Thermo Fisher Scientific), insulin-transferrin-selenite (#41400045; Thermo Fisher Scientific), non-essential amino acids (#11140050; Thermo Fisher Scientific), Glutamax (#35050061; Thermo Fisher Scientific), human insulin (#I2643-25 mg; Sigma) and monothioglycerol (#M1753; Sigma) supplemented with 25 ng/ml human M-CSF (#PHC9501; Thermo Fisher Scientific), 50 ng/ml TGF-β1 (#130-108-969; Miltenyl), and 100 ng/ml IL-34 (#200-34; Peprotech). After 24 days in this medium, two additional cytokines, 100 ng/ml CD200 (#C311; NovoProtein), and 100 ng/ml CX3CL1 (#300-31; PeproTech), were added to the medium described above to mature MGLs. MGLs were cultured in this new medium for an additional week and then harvested for experiments.

### Drug Treatments

MGLs generated from WT/WT and ΔE9/ΔE9 hiPSCs were plated into a 48-well plate coated with poly-L-lysine (# P6282; Sigma) at a cell density of 200,000 cells/well. After 24 h, MGLs were treated with either DMSO (#D8418; Sigma) or lipopolysaccharides (LPS, 100 ng/ml) (#4391; Sigma) with or without a casein kinase inhibitor, CX-4945 (#A8330; ApexBio Technology; 0.5 μM) or SGC-CK2-1 (Axtman lab; 10 nM), and cultured for 24 h. At the end of 24 h, RNA, protein, and condidtioned medium were harvested from the MGLs.

### Toxicity Analysis

MGLs generated from WT hiPSCs were plated as described above. Increasing doses of either SGC-CK2-1 (10–100 μM) or CX-4945 (500–100 μM) were applied to the cells for 24 h. Cell toxicity was analyzed by calculating the percent live cells using Trypan Blue.

### Reverse Transcriptase-Polymerase Chain Reaction (RT-PCR)

For mRNA expression analysis, RNA was extracted using Trizol (#15596018; Thermo Fisher Scientific) and first strand cDNA synthesis was performed using the iScript cDNA Synthesis kit (#1708890; Bio-Rad). Quantitative RT-PCR (qRT-PCR) was performed with SYBR Green Master Mix (#A46012; Thermo Fisher Scientific). Results were quantified using the Delta-Delta Ct method (Livak and Schmittgen, 2001). qRT-PCR was performed with the following primers—IL-6 forward primer 5′ TGCAATAACCACCCCTGACC 3′ and reverse primer 5′ TGCGCAGAATGAGATGAGTTG 3′; IL-1β forward primer 5′ TTTGAGTCTGCCCAGTTCCC 3′ and reverse primer 5′ TCAGTTATATCCTGGCCGCC 3′; RPL13 forward primer 5′AGCCTACAAGAAAGTTTGCCTAT 3′ and reverse primer 5′ TCTTCTTCCGGTAGTGGATCTTGGC 3′; CYC1 forward primer 5′ CCTGGAGGAGAAGAGGAAAGAGA 3′ and reverse primer 5′ TTGAGGACCTCTGTGTATTTGTCAA 3′. IL-6 and IL-1β values were normalized to the geometric mean of the housekeeping genes, RPL13 and CYC1. qRT-PCR results were calculated using the 2^–ΔΔ^*^CT^* method and presented as fold change from DMSO following the procedure described by Rao et al. (2013).

### Enzyme-Linked Immunosorbent Assays

To measure IL-6 and IL-1β secretion, ELISAs were performed for both cytokines using manufacturer’s instructions (IL-6: ab178013, abcam; IL-1β: ab214025, abcam). Briefly, MGLs were plated onto poly-l-lysine coated 96 well plates, treated with DMSO, LPS, SGC-CK2-1, CX-4945, SGC-CK2-1+LPS and CX-4945+LPS for 24 h and media was harvested at the end of 24 h. Colorimetric assays were performed using ELISA kits described above and optical density was measured using Perkin Elmer/Wallac Envision 2100 Multilabel Reader.

### Western Blot Analysis

Western blot analysis was performed under denaturing conditions using 4–15% gradient polyacrylamide gels (Bio-Rad, #4561084). Proteins were electroblotted onto PVDF membrane (Bio-Rad, #1620177), fixed with 0.4% paraformaldehyde for 30 min and blocked in a blocking buffer containing 5% nonfat dry milk before incubating at 4°C overnight with primary antibody [AKT (pan) (40D4) antibody, Cell Signaling #2920, 1:1,000 dilution, AKT1 (phospho S129) antibody (EPR6150), abcam #133458, 1:1,000 dilution = 1.8 μg/ml] and β-actin (clone AC-15) (Millipore Sigma A5441), 1:7,500 dilution, diluted in 5% bovine serum albumin and 0.2% NaN_3_. Membranes were washed in phosphate-buffered saline with 0.05% Tween 20 (PBST) and incubated with appropriate horseradish peroxidase-conjugated secondary antibody (1:7500; GE Healthcare, #NA931V, #NA934V) diluted in the blocking buffer. After rinses with PBST, membranes were developed with Clarity Max ECL Western Blotting Substrate (Bio-Rad, #1705062) and exposed to CL-XPosure Film (Thermo Fisher Scientific, #34091). Quantification of Western blots was performed using ImageJ software.

### Statistical Analysis

For all experiments presented in the study, each cell line was differentiated three times and the data represents three biological replicates with 2–3 technical replicates per experiment.

Data points were analyzed for normal distributions and either a one-way ANOVA analysis or a paired *t*-test was performed using GraphPad Prism. Error bars indicate Standard Deviations.

## Results

### Selection of Casein Kinase 2 Inhibitors and Dosing Based Upon Published Data

**FIGURE 1:**
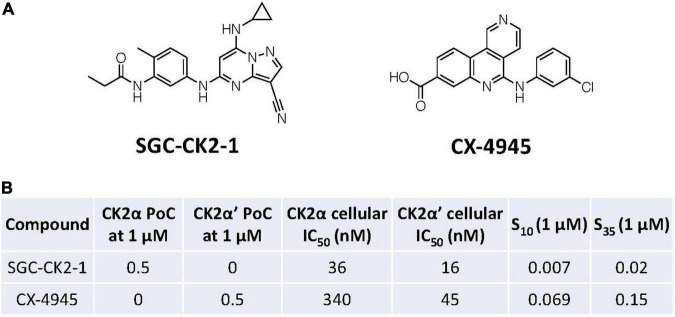
Summary of CK2 inhibitor data. **(A)** Structures of SGC-CK2-1 and CX-4945. **(B)** Table of published kinome-wide selectivity scores (S_10_ and S_35_), CK2α and CK20’ binding assay data (PoC at 1 μM), and CK2α and CK2α’ cellular target engagement (NanoBRET) IC_50_ values for SGC-CK2-1 and CX-4945.

**FIGURE 2:**
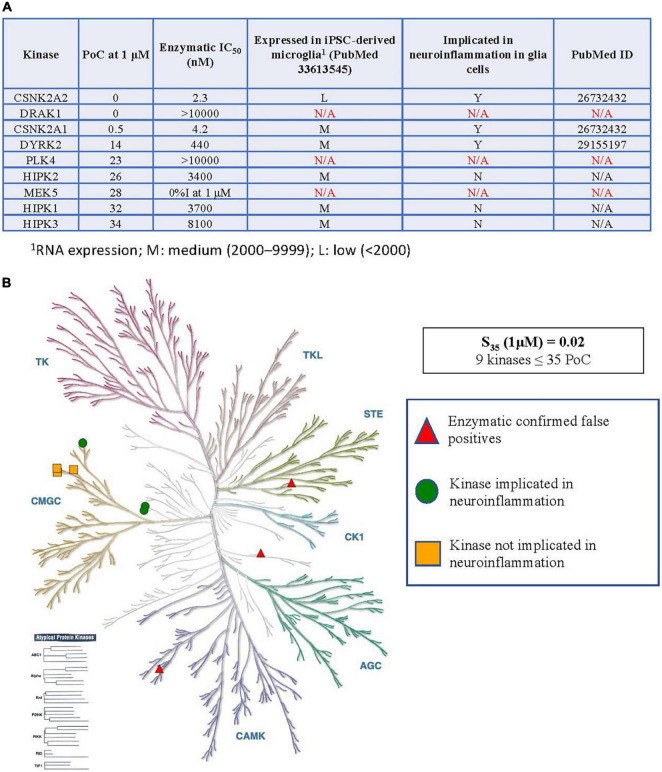
Kinases inhibited by SGC-CK2-1 implicated in neuroinflammation. **(A)** Table of S_35_ kinases, corresponding enzymatic follow-up, expression of inhibited kinases (minus confirmed false positives) in microglia, and reported connections to neuroinflammation in glial cells. **(B)** Visual representation of the distribution of S_35_ kinases around the kinome tree. ^1^RNA expression; M, medium (2,000–9,999); L, low (<2,000).

**FIGURE 3:**
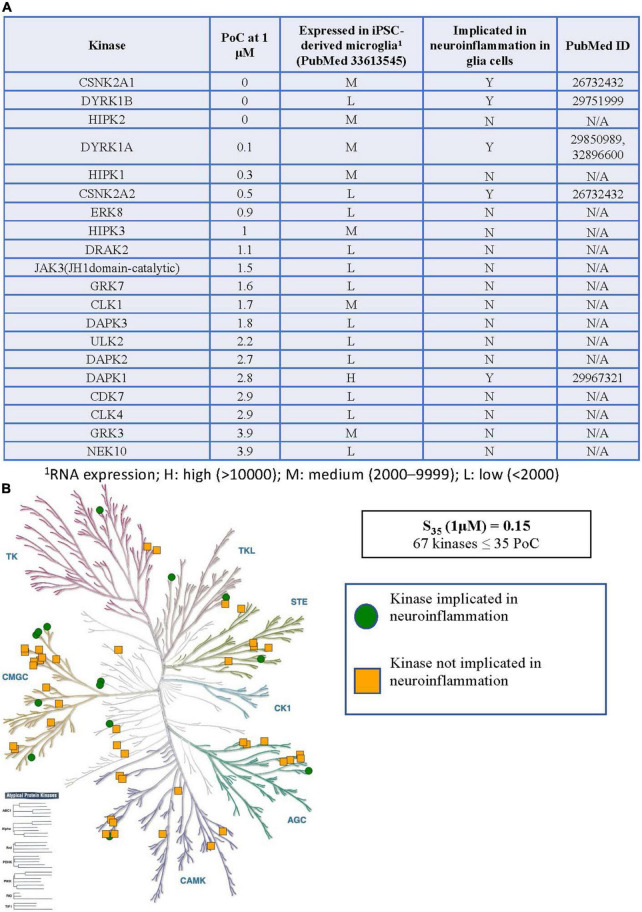
Kinases inhibited by CX-4945 implicated in neuroinflammation. **(A)** Table of S_35_ kinases with PoC < 5, expression of inhibited kinases in microglia, and reported connections to neuroinflammation in glial cells. **(B)** Visual representation of the distribution of all S_35_ kinases around the kinome tree. ^1^RNA expression; H, high (>10,000); M, medium (2,000–9,999); L, low (<2,000).

Two CK2 inhibitors (Figure 1) were chosen for study based upon literature reports detailing their potent inhibition of this enzyme. These two inhibitors, however, are quite different when considering their potency and kinome-wide selectivity. CX-4945, one of the most employed CK2 inhibitors in published studies and an advanced agent in clinical trials, demonstrates sub-optimal kinome-wide selectivity, significantly inhibiting several kinases with IC_50_ values < 100 nM (Pierre et al., 2011; Wells et al., 2021). The table in Figure 1B summarizes the published potency and selectivity data associated with CX-4945 vs. recently disclosed CK2 chemical probe, SGC-CK2-1 (Wells et al., 2021). For reference, the S_10_(1 μM) selectivity scores reported in Figure 1B were calculated by dividing the number of WT human kinases that exhibit percent of control (PoC) < 10 by the total number of WT human kinases that exhibit > 90% inhibition by the total number of WT human kinases after dosing with 1 μM of the given inhibitor and profiling in the DiscoverX *scan*MAX panel. In the same manner, an S_35_(1 μM) score is representative of the percentage of WT human kinases with PoC < 35 after dosing with 1 μM of the given inhibitor and profiling in the DiscoverX *scan*MAX panel. Accordingly, lower S_10_(1 μM) or S_35_(1 μM) values indicate high compound selectivity (Bosc et al., 2017). PoC values reported for various kinases in Figures 1–3 and Supplementary Table 1 were generated *via* screening at 1 μM in the DiscoverX *scan*MAX panel (Wells et al., 2021). Lower PoC values are indicative of more efficacious binding of the inhibitor to that particular kinase. Doses of CX-4945 and SGC-CK2-1 were selected based on the data in Figure 1, considering both the CK2 cellular IC_50_ values as well as the kinome-wide selectivity at 1 μM (Figures 1–3). In addition, the dosing window used in a previous study that explored the impact of treatment with CX-4945 on cytokine secretion in astrocytes was considered (Rosenberger et al., 2016). Doses above and below the CK2 cellular IC_50_ values for SGC-CK2-1 were probed, offering the ability to inhibit only CK2 and no other kinases. As previously discussed, the kinase next most potently inhibited by SGC-CK2-1 is DYRK2, which has a cellular IC_50_ value = 3,700 nM when evaluated using the same assay format (Wells et al., 2021). Doses of CX-4945 were selected to remain near or below the 1 μM concentration at which the kinome-wide selectivity was profiled, knowing that CK2 would be potently inhibited given the cellular IC_50_ values for this compound. Inhibition of downstream signaling of CK2 in cells has been reported when using our selected doses of each inhibitor (Siddiqui-Jain et al., 2010; Wells et al., 2021).

### Summary of Kinases Inhibited by SGC-CK2-1 Implicated in Neuroinflammation

As kinases are known to regulate many signaling pathways and play divergent roles, each kinase inhibited within the S_35_(1 μM) fraction by CK2 compounds in Figure 1 was explored in more detail. The table in Figure 2A shows all kinases with PoC ≤ 35 in the DiscoverX *scan*MAX panel when SGC-CK2-1 was dosed at 1 μM (Wells et al., 2021). Enzymatic follow-up, carried out at the K_*m*_ = ATP for each respective kinase, identified 3 kinases as false positives: DRAK1, PLK4, and MEK5 (Wells et al., 2021). These kinases were not considered further since it was likely that they were artifacts of the binding assay and not inhibited by SGC-CK2-1 (indicated with N/A in Figure 2A). To determine the relevance of using hiPSC-derived microglia to test this selective probe, we analyzed RNA-seq data from microglia-like cells generated in monoculture from hiPSCs (GEO accession GSE159108) (Reich et al., 2020). We considered the expression of these S_35_ kinases and expression was categorized as low: (L, < 2,000), medium (M, 2,000–9999), or high (H, > 10,000) based on the bulk RNA-seq values, which were averaged across 5 independent samples. A literature survey was then employed to determine whether a role in neuroinflammation for each of these kinases has been described specifically in glial cells, citing the PubMed source of the data in the table as well. All nine S_35_ kinases were then plotted on a kinome tree (Figure 2B). The data in Figure 2 supports that expression of all kinases inhibited by SGC-CK2-1 is low to medium in WT hiPSC-derived microglia.

### Summary of Kinases Inhibited by CX-4945 Implicated in Neuroinflammation

When considering the inhibitor CX-4945, the situation becomes much more complex. The table in Figure 3A shows all WT kinases with PoC < 5 in the DiscoverX *scan*MAX panel when CX-4945 was dosed at 1 μM (Wells et al., 2021). This represents the fraction of kinases most potently inhibited by CX-4945. All 59 WT, non-mutant kinases with PoC ≤ 35 are included in Supplementary Table 1. Given the number of kinases with PoC ≤ 35, enzymatic follow-up has not been carried out to confirm or refute inhibition of any of these kinases by CX-4945. Orthogonal profiling in other CX-4945 publications offers validation of inhibition of CLK1–CLK3, DAPK3, DYRK1A, DYRK1B, HIPK3, PIM1, and TBK1 (Pierre et al., 2011; Kim et al., 2014, 2016). The same RNAseq data from hiPSC-derived microglia-like cells that were generated in monoculture from GEO accession GSE159108 (Reich et al., 2020) was next consulted to categorize the expression of the 59 WT S_35_ kinases as low (L), medium (M), or high (H) as defined in the previous section. Published connections of these kinases to glia-mediated neuroinflammation and relevant references were also noted in Figure 3A and Supplementary Table 1. The tree diagram in Figure 3B excludes three lipid kinases and RSK1 (two domains listed in Supplementary Table 1) has only been plotted once, showing the distribution of the remaining 56 S_35_ kinases for CX-4945.

### Low Nanomolar Doses of SGC-CK2-1 Blunt an Inflammatory Response in Human Induced Pluripotent Stem Cells-Derived Microglial Like Cells Without Associated Toxicity and Suppress Inflammatory Cytokine Expression More Efficiently Than CX-4945

We directly tested whether SGC-CK2-1 could inhibit an inflammatory response more efficiently than CX-4945 using hiPSC-derived MGLs stimulated with LPS. In these experiments we differentiated WT hiPSCs or hiPSCs engineered to harbor two copies of a FAD mutation in PSEN1, PS1ΔE9/ΔE9, into MGLs using published protocols (McQuade et al., 2018). The aim of these experiments was not to draw conclusion about the genetic background of the cells, but to perform a proof-of-concept experiment showing that either WT or AD related cells respond to this type of treatment, which is important for future small molecule screens.

**FIGURE 4:**
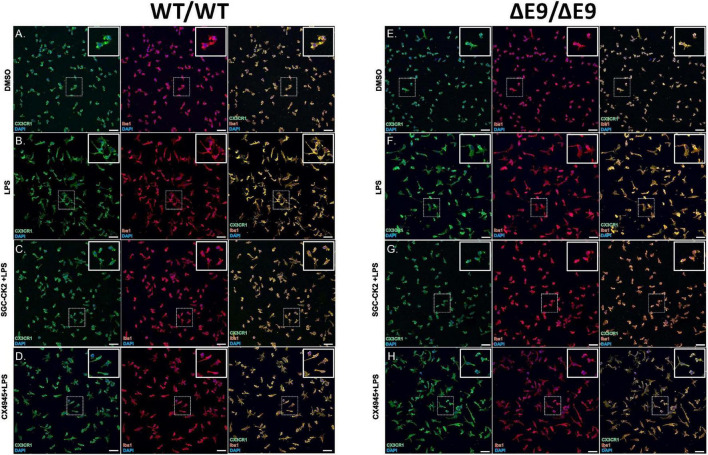
Characterization of DMSO, LPS and drug treated MGLs derived from WT/WT and ΔE9/ΔE9 (FAD) iPSCs. WT **(A–D)** and FAD **(E–H)** MGLs were stimulated with LPS **(B,F)** and simultaneously treated with either SGC-CK2-1 **(C,G)** or CX-4945 **(D,H)** for 24 h and immunocytochemistry was performed. In all conditions, MGLs robustly express microglia specific markers, CX3CR1 and Iba1. Scale bar = 50 μm.

First, we characterized MGLs derived from both of these cell lines and noted no differences in microglial markers in these cells either under baseline or stimulated conditions (Figures 4A–H), although the morphology of the cells in both cell lines was altered when treated with LPS (Figures 4B,F). Next, we performed a dose-response experiment in WT MGLs and found that SGC-CK2-1 was toxic at concentrations higher than 1 μM (Supplementary Figure 1A). Treatment of WT MGLs with LPS significantly induced mRNA expression of pro-inflammatory cytokine genes IL-6 and IL1-β and this expression was significantly reduced at both the 10 and 100 nM concentrations of SGC-CK2-1 (Supplementary Figure 1B). Recent literature (D’Amore et al., 2020) suggested an optimal concentration of CX-4945 at 500 nM. This concentration was also not toxic to hiPSC-MGLs, although toxicity was observed at concentrations greater than 1 μM for this compound (Supplementary Figure 1C). To confirm that 10 nM SGC-CK2-1 was effectively inhibiting kinase activity in MGLs, we analyzed phosphorylated AKT (Ser 129), a validated substrate of CK2 (Di Maira et al., 2005). We confirmed that CK2 is inhibited by documenting a 50% reduction in a phosphorylated AKT (Ser 129), as compared to total AKT, in microglia cultures treated with 10 nM SGC-CK2-1 (Supplementary Figure 1D).

**FIGURE 5:**
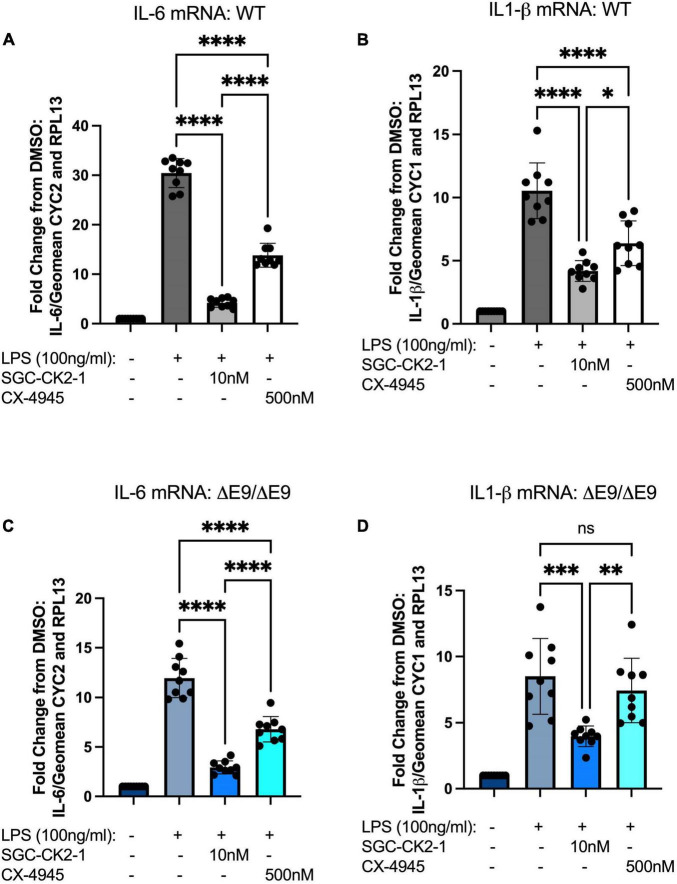
SGC-CK2-1 is more effective at inhibiting mRNA expression of IL-6 and IL1-β in hiPSC-MGLs after LPS stimulation than CX-4945. WT **(A,B)** and FAD **(C,D)** MGLs were stimulated with LPS and simultaneously treated with either SGC-CK2-1 or CX-4945 for 24 h. In all conditions, SGC-CK2-1 was more effective at inhibiting both IL-6 and IL1-β mRNA than CX-4945. These data represent three independent differentiations. ^****^p ≤ 0.0001; ^***^p = 0.0006; ^**^p = 0.0077; *p = 0.0293; ns, not significant by one-way ANOVA analysis. Error bars indicate the mean ± SD.

**FIGURE 6:**
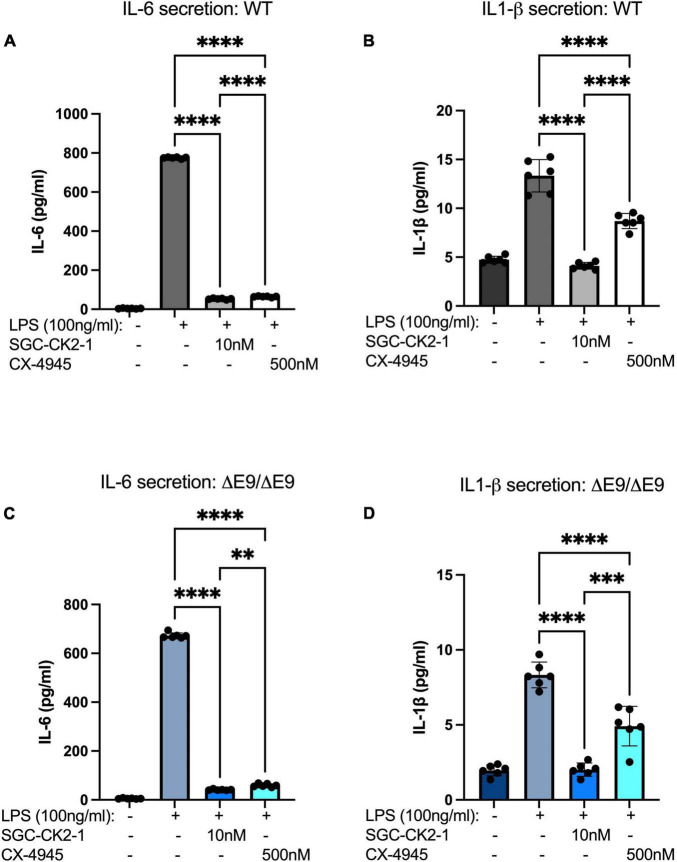
SGC-CK2-1 reduces pro-inflammatory cytokine secretion more effectively than CX-4945. WT **(A,B)** and FAD **(C,D)** MGLs were stimulated with LPS and simultaneously treated with either SGC-CK2-1 or CX-4945 for 24 h. In all conditions, SGC-CK2-1 was more effective at inhibiting both IL-6 and IL1-β cytokine secretion into the culture media than CX-4945. These data represent three independent differentiations. ^****^p ≤ 0.0001; ^***^p = 0.0006; ^**^p = 0.0024 by one-way ANOVA analysis. Error bars indicate the mean ± SD.

We next wanted to directly compare the efficacy of SGC-CK2-1 with CX-4945. We performed three independent differentiations of both WT and PS1ΔE9/ΔE9 hiPSC to MGLs, stimulated with LPS and treated with 10 nM SGC-CK2-1 (selected based on results in Supplementary Figures 1A,B) or 500 nM of CX-4945, which did not show detectable cytotoxicity (Supplementary Figure 1C). In both WT and PS1ΔE9/ΔE9 MGLs we observed a significant induction of mRNA expression of IL-6 and IL1-β after LPS treatment (Figure 5). In both cell lines, SCG-CK2-1 blunted mRNA induction of these cytokines significantly more than CX-4945. Interestingly, with CX-4945 treatment, mRNA expression of IL1-β in PS1ΔE9/ΔE9 MGLs was not significantly reduced (Figure 5D). These results were consistent across the three independent differentiations for each cell line. Since the 50-fold higher dose of CX-4945 could not recapitulate the result observed with 10 nM of SGC-CK2-1, responses to lower concentrations of CX-4945 were not explored. We next analyzed the release of IL-6 and IL-1β by PS1ΔE9/ΔE9 MGLs into the culture medium using an ELISA assay. A significant increase in cytokine protein released into the culture medium in MGLs treated with LPS (Figures 6A–D). This increase is ameliorated by treatment with the inhibitors and the reduction by SGC-CK2-1 is more significant than with the commercial molecule CX-4945 (Figures 6A–D). Interestingly, at the protein level, we noticed a significant reduction of secreted IL-6 and IL-β in both WT and PS1ΔE9/ΔE9 MGLs, which is somewhat discordant with our mRNA results. However, the timing of mRNA induction and subsequent protein translation, secretion, and/or turn-over is known to vary and to not always be correlative (Liu et al., 2016; Moritz et al., 2019). Thus, it is important to explore orthogonal measurements of both gene and protein expression.

## Discussion

Alzheimer’s disease is a complex neurodegenerative disorder. The failure of most clinical trials targeting Aβ in AD suggests that it is imperative to look at other pathways in the central nervous system that drive neurodegeneration. One challenge for AD, and other age-related neurodegenerative disorders, is that relevant tissue only becomes available at the end stage of a long and progressive disease. hiPSC models can help to mitigate this challenge as living, relevant human cell types can be generated *in vitro* and recapitulate the genetics and cellular phenotypes of the disorder (Penney et al., 2020). hiPSC-derived cells are emerging as powerful pre-clinical models because potential therapeutics can be screened in human cell types and in a high-throughput manner. Recent studies have used hiPSC-derived neural cells to look at small molecules that modulate Aβ, tau, and synaptic activity (Trudler et al., 2021). However, many of these studies focus on neuron-specific read-outs. Here, we employ hiPSC-derived MGLs to test a specific chemical probe that targets the protein kinase CK2 and look at proinflammatory cytokine expression as a read-out of microglia-mediated neuroinflammation.

With a collection of more than 300 known substrates, it is not surprising that several inflammatory mediators are phosphorylated by CK2. NF-κB, STAT1, STAT3, CREB, CREM, Sp1, and C/EBP are examples of transcription factors regulated by CK2 with a role in inflammation and associated disorders (Bird et al., 1997; Yamaguchi et al., 1998; Filhol et al., 2004; Singh and Ramji, 2008; Drygin et al., 2011). NF-κB regulation by CK2 has been extensively characterized in epithelial, HeLa, and diploid gingival fibroblast cells (Bird et al., 1997; Wang et al., 2000; Parhar et al., 2007; Singh and Ramji, 2008). Furthermore, CK2 activity is stimulated following treatment with proinflammatory agents (LPS) or cytokines (TNF-α, IL-1, TGF-β, or IFN-γ) in a variety of murine and human cell types (Lodie et al., 1997; Rosenberger et al., 2016). These stimuli activate and/or increase the expression of CK2 and/or increase its association with downstream targets or other signaling proteins. Signaling *via* key transcription factors is increased in response, leading to the elevated expression of downstream cytokines and other inflammatory mediators (Singh and Ramji, 2008). The fact that there are multiple CK2 targets that mediate inflammation should increase the efficacy of CK2 inhibitors as anti-inflammatory agents. Unraveling the complex regulation of CK2 signaling and its roles in response to various stimuli that mediate inflammation will be essential if CK2 is to be pursued therapeutically for diseases with an associated inflammatory component, like AD. Also, since CK2 plays a critical role in normal cells, development of targeting strategies that enable specific inhibition of its action in cells responsible for mediating inflammation to drive disease pathology represents an added challenge.

Outside of the brain, CK2 has been shown to contribute to chronic inflammation in non-neuronal cell types including breast cancer, intestinal, renal, immune, and myeloid cells (Yamada et al., 2005; Parhar et al., 2007; Drygin et al., 2011; Koch et al., 2013; Larson et al., 2020). Aberrant CK2 signaling in these cells is associated with inflammatory diseases such as breast cancer, glomerulonephritis, autoimmune encephalomyelitis, atherosclerosis, and T cell lymphoma (Yamaguchi et al., 1998). Additionally, CK2 is known to partially mediate the switch to a more pro-inflammatory state in mouse macrophages (Hagemann et al., 2008; Chen et al., 2017). Inhibition of CK2 decreases secretion of IL-6 in breast cancer cells (Drygin et al., 2011) and mouse macrophages (Glushkova et al., 2018). CK2 is also highly expressed in the brain and CK2 protein levels are reportedly elevated in the AD brain (Rosenberger et al., 2016). Within the AD brain, CK2 localizes to astrocytes associated with cerebral amyloid angiopathy. These astrocytes function during the neuroinflammatory response in AD and CK2 plays a role in this process (Rosenberger et al., 2016). However, whether CK2 mediates a similar inflammatory response in microglia, innate immune cells of the brain derived from a myeloid lineage, is not known.

When considering the narrow kinase inhibition profile of SGC-CK2-1, in addition to CK2, only DYRK2 has an associated neuroinflammatory phenotype described in glial cells (Figure 2A). It is worth noting, however, that HIPK family kinases are not very well studied (HIPK1 and HIPK3 are on the NIH-nominated list of understudied kinases defined by the Illuminating the Druggable Genome (IDG) program (Berginski et al., 2020), and it is therefore possible that these kinases could have a role in neuroinflammation that has not yet been characterized. We also acknowledge that pertinent references may have been missed in our literature survey. In terms of CK2- vs. DYRK2-mediated activity, there is a large (100-fold) dosing window that enables inhibition of CK2 without eliciting inhibition of DYRK2 if a proper concentration is selected. Our dose of 10 nM for studies herein was selected to enable inhibition of CK2 without off-target DYRK2 inhibition. The narrow kinome-wide profile of SGC-CK2-1 makes it an ideal candidate molecule to use to interrogate the role of CK2 in neuroinflammation.

With respect to CX-4945, many of the S_35_ kinases with low expression in WT hiPSC-derived microglia correspondingly do not have an associated neuroinflammatory phenotype in glia, while several with medium or high expression have a described role as a mediator of neuroinflammation (Figure 3A and Supplementary Table 1). This correlation suggests that significant expression of a kinase is one factor that contributes to it having a pronounced role in mediating glial neuroinflammatory processes. As was discussed with respect to the HIPK family and SGC-CK2-1, many kinases inhibited in the S_35_ fraction by CX-4945 are not well studied. In fact, 21 were on the original IDG list of understudied kinases (Berginski et al., 2020). The role of these kinases in mediating neuroinflammation could be delineated if they receive additional attention and high-quality chemical tools are developed to enable their study. As support of this hypothesis, CSNK2A2, CDKL5, DYRK1B, and DYRK2 were the only four IDG kinases on the full list of S_35_ kinases (Supplementary Table 1) with an associated neuroinflammatory role described in the literature. In total, thirteen kinases in addition to CK2 that are potently inhibited by CX-4945 have been described as mediators of neuroinflammatory responses in glia. With so many possible off-target kinases responsible for the response observed when treating with CX-4945, one cannot reliably ascribe a phenotype to CK2 when dosing with CX-4945.

Preclinical studies to test small molecules that may be effective against neurodegeneration in AD and other disorders in animal models or transformed cell lines are informative but can miss important human-specific genetics that influence phenotypes and important cell-type specific differences may impact the effects of a particular drug. hiPSCs are a promising model to overcome some of these limitations. hiPSCs are now efficiently differentiated into all cell types of the CNS (Penney et al., 2020) and advances in 3-D modeling using cerebral organoids have enabled using these to test small molecules in a high-throughput manner (Costamagna et al., 2021; Park et al., 2021). However, in general, many recent studies involving small molecule screening of AD phenotypes using hiPSC models have been performed in neurons (Yahata et al., 2011; Brownjohn et al., 2017; Kondo et al., 2017; van der Kant et al., 2019). Here, we aimed to perform a side-by-side test of a selective and a non-selective kinase inhibitor using hiPSC-derived MGLs and probe a neuroinflammatory response. Our data encompasses both a WT and a familial AD genetic background and shows that the selective inhibitor SGC-CK2-1 efficiently inhibits an LPS-induced inflammatory response in both cell lines with consistent results between independent differentiations. Furthermore, this inhibitor demonstrates a more effective reduction in pro-inflammatory cytokine expression and secretion than CX-4945 (Figures 5, 6). Our study is not powered to directly analyze molecular links between CK2 and PSEN1 mutations. It is certainly plausible that different FAD mutations and genetic variants linked to increased AD risk may modify the inhibitory effects of SGC-CK2-1. However, together our data suggest that this small molecule could be broadly effective across both strongly genetic and sporadic AD cases.

Our study demonstrates the benefits of side-by-side testing of selective vs. non-selective inhibitors in a relevant cell type. Future work that encompasses side-by-side testing in other CNS cell types and assaying for cell-type specific phenotypes (i.e., tau phosphorylation in neurons or cholesterol transport in astrocytes) will certainly be informative. In addition, our study does not have the power to detect intrinsic differences in the inflammatory response based on cell line genotype. Therefore, additional studies aimed at analyzing multiple cell lines with AD mutations or risk variants compared with isogenic wild-type cells will be necessary to understand the biological mechanisms behind the neuroinflammatory response in these cells. Future work can include incorporating humanized cells into mouse models to further test the effects of these compounds in an *in vivo* model.

In general, our work and multiple other studies in the field, have highlighted the utility of hiPSC-derived cells as tools for pre-clinical study *in vitro*, especially related to evaluation of high-quality chemical tools. The hiPSC-derived model is a powerful methodology to simultaneously begin to validate therapeutic hypotheses in a relevant cellular system and identify potential chemical starting points for drug development. Evaluation of chemical probes in hiPSC-derived cellular systems has the potential to illuminate new opportunities for therapeutic intervention for diseases of the CNS and could prove to be transformative.

## Conclusion

Our study has certain limitations. First, we have performed this as proof-of-concept and used only one cell line that is representative of an autosomal dominant AD background and one cell line that is representative of a wild-type background. The goal of our experiments was to directly test a chemical probe vs. a commercial inhibitor. There are many future studies needed to understand how CK2 inhibition may impact the full range of pathologies present in AD. However, in this work, we show that SGC-CK2-1 is a potent suppressor of the CK2-mediated neuroinflammatory response and outperforms CX-4945 in inhibiting the expression of the proinflammatory cytokines IL-6 and IL-1β. These results could partially be explained by previous work showing that SGC-CK2-1 is both more potent and more selective for CK2 than CX-4945 when tested in cancer cell lines, but also provide new data underscoring the power associated with profiling selective probes in an AD-relevant cell type and model.

## Data Availability Statement

The original contributions presented in this study are included in the article/Supplementary Material, further inquiries can be directed to the corresponding author/s.

## Author Contributions

SM, ADA, and JY conceived to the project and contributed to the writing and editing of the manuscript. ADA performed the DiscoverX *scan*MAX analysis, analysis of bulk-RNAseq, and generated the kinome trees *via* KinMap (Eid et al., 2017). SM and CK performed all hiPSC-MGLs experiments. SM and JY analyzed the hiPSC-MGLs experiments. All authors contributed to the article and approved the submitted version.

## Conflict of Interest

The authors declare that the research was conducted in the absence of any commercial or financial relationships that could be construed as a potential conflict of interest.

## Publisher’s Note

All claims expressed in this article are solely those of the authors and do not necessarily represent those of their affiliated organizations, or those of the publisher, the editors and the reviewers. Any product that may be evaluated in this article, or claim that may be made by its manufacturer, is not guaranteed or endorsed by the publisher.
