# Erratum: Evaluation of a selective chemical probe validates that CK2 mediates neuroinflammation in a human induced pluripotent stem cell-derived microglial model

**DOI:** 10.3389/fnmol.2023.1334040

**Published:** 2023-11-21

**Authors:** Frontiers Production Office

**Affiliations:** Frontiers Media SA, Lausanne, Switzerland

**Keywords:** Alzheimer's disease, hiPSC models, casein kinase 2, neuroinflammation, chemical probe, SGC-CK2-1

Due to a production error, the word “microglial” was misspelled as “mircroglial” in the article title.

The publisher apologizes for this mistake. The original article has been updated.

