# Supplementary figures and images for: Evaluation of a Selective Chemical Probe Validates That CK2 Mediates Neuroinflammation in a Human Induced Pluripotent Stem Cell-Derived Microglial Model
Source: Front Mol Neurosci. 2022 Jun 14;15:824956. doi: 10.3389/fnmol.2022.824956 (PMC9239073; doi:10.3389/fnmol.2022.824956)

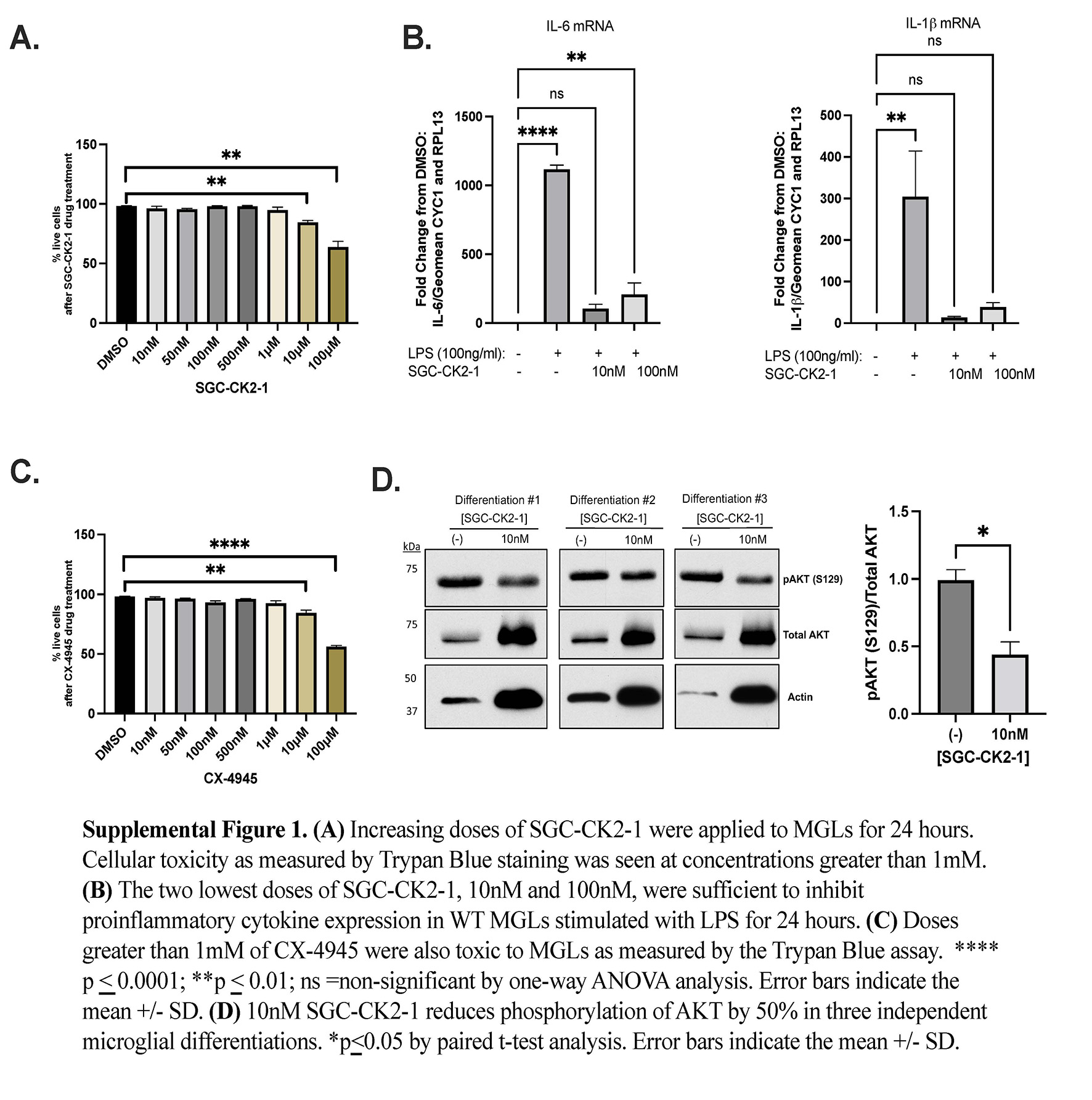

Supplement: Supplementary file 1 [file Image_1.JPEG]
