# Supplementary material for: Evaluation of a Selective Chemical Probe Validates That CK2 Mediates Neuroinflammation in a Human Induced Pluripotent Stem Cell-Derived Microglial Model
Source: Front Mol Neurosci. 2022 Jun 14;15:824956. doi: 10.3389/fnmol.2022.824956 (PMC9239073; doi:10.3389/fnmol.2022.824956)
Supplement: Supplementary file 2 [file Table_1.pdf]

Kinase	PoC at 1 μM	Expressed in iPSC-derived microglia[1] (PubMed 33613545)	Implicated in neuroinflammation in glia cells	PubMed ID
CSNK2A1	0	M	Y	26732432
DYRK1B	0	L	Y	29751999
HIPK2	0	M	N	N/A
DYRK1A	0.1	M	Y	29850989, 32896600
HIPK1	0.3	M	N	N/A
CSNK2A2	0.5	L	Y	26732432
ERK8	0.9	L	N	N/A
HIPK3	1	M	N	N/A
DRAK2	1.1	L	N	N/A
JAK3(JH1domain-catalytic)	1.5	L	N	N/A
GRK7	1.6	L	N	N/A
CLK1	1.7	M	N	N/A
DAPK3	1.8	L	N	N/A
ULK2	2.2	L	N	N/A
DAPK2	2.7	L	N	N/A
DAPK1	2.8	H	Y	29967321
CDK7	2.9	L	N	N/A
CLK4	2.9	L	N	N/A
GRK3	3.9	M	N	N/A
NEK10	3.9	L	N	N/A
DYRK2	5.1	M	Y	29155197
YSK4	5.4	L	N	N/A
PIM1	5.8	M	N	N/A
CLK2	6.5	M	N	N/A
PIM3	7.1	M	N	N/A
HIPK4	7.8	L	N	N/A
TYK2(JH2domain-pseudokinase)	9.4	H	N	N/A
RSK3(Kin.Dom.2-C-terminal)	9.8	L	N	N/A
DRAK1	10	L	N	N/A
ICK	10	L	N	N/A
IRAK1	11	H	Y	31883536
TAOK1	11	M	N	N/A
GRK1	14	L	N	N/A
PIKFYVE	16	M	N	N/A
PFTK1	17	L	N	N/A
CDKL5	18	L	Y	34238328
GAK	19	M	N	N/A
MEK5	19	L	N	N/A
STK33	21	L	N	N/A
RSK2(Kin.Dom.1-N-terminal)	23	M	N	N/A
RSK4(Kin.Dom.1-N-terminal)	23	L	N	N/A
CLK3	25	M	N	N/A
GCN2(Kin.Dom.2,S808G)	26	M	N	N/A
TBK1	26	M	Y	27211305, 30635357
BMPR2	27	M	N	N/A
PIP5K2C	28	M	N	N/A
CDK5	29	M	Y	22262900, 24495352
LOK	29	H	N	N/A
PAK2	29	H	Y	29129728
RSK1(Kin.Dom.1-N-terminal)	29	H	N	N/A
MELK	30	L	N	N/A
PFTAIRE2	30	L	N	N/A
SGK	31	H	Y	33646633, 29755644
PHKG2	32	M	N	N/A
RSK1(Kin.Dom.2-C-terminal)	32	H	N	N/A
SLK	32	M	N	N/A
DCAMKL3	34	L	N	N/A
MERTK	35	H	Y	28500071, 28925029
PIK3CG	35	M	Y	20025958
TGFBR2	35	H	Y	31727112

Supplemental Table 1. All kinases with PoC ≤ 35 inhibited by CX-4945, expression of inhibited kinases in microglia, and reported connections to neuroinflammation in glial cells.
